# Structural Insights into Ring Formation of Cohesin and Related Smc Complexes

**DOI:** 10.1016/j.tcb.2016.04.002

**Published:** 2016-09

**Authors:** Thomas Gligoris, Jan Löwe

**Affiliations:** 1 Department of Biochemistry, University of Oxford, Oxford, OX1 3QU, UK; 2 MRC Laboratory of Molecular Biology, Cambridge, CB2 0QH, UK

## Abstract

Cohesin facilitates sister chromatid cohesion through the formation of a large ring structure that encircles DNA. Its function relies on two structural maintenance of chromosomes (Smc) proteins, which are found in almost all organisms tested, from bacteria to humans. In accordance with their ubiquity, Smc complexes, such as cohesin, condensin, Smc5-6, and the dosage compensation complex, affect almost all processes of DNA homeostasis. Although their precise molecular mechanism remains enigmatic, here we provide an overview of the architecture of eukaryotic Smc complexes with a particular focus on cohesin, which has seen the most progress recently. Given the evident conservation of many structural features between Smc complexes, it is expected that architecture and topology will have a significant role when deciphering their precise molecular mechanisms.

## Trends

SMC dimeric complexes combine with a subunit of the kleisin family and form ring complexes entrapping DNA. In eukaryotes, cohesins, condensins, and SMC5-6 act on sister chromatid cohesion, chromosome condensation, and DNA repair.

Cohesin complexes are dynamic as a result of two opposing activities: a DNA-loading activity mediated by the Scc2-Scc4 complex and a releasing activity elicited by the Pds5-Wapl-Scc3 complex. Crystal structures of these proteins are providing insight into their function. Pds5 and Scc3 have distinct positive functions in cohesion maintenance after S phase. The concept emerging is that different arrangements of these complexes exist in different stages of the cell cycle.

SMCs are found in both open V-shaped and closed rod-shaped forms, which likely represent functional states of the complexes. Their formation depends on interactions with DNA and ATP hydrolysis.

## General Features of Smc Proteins and Classes of Smc Kleisins

**Figure 1:**
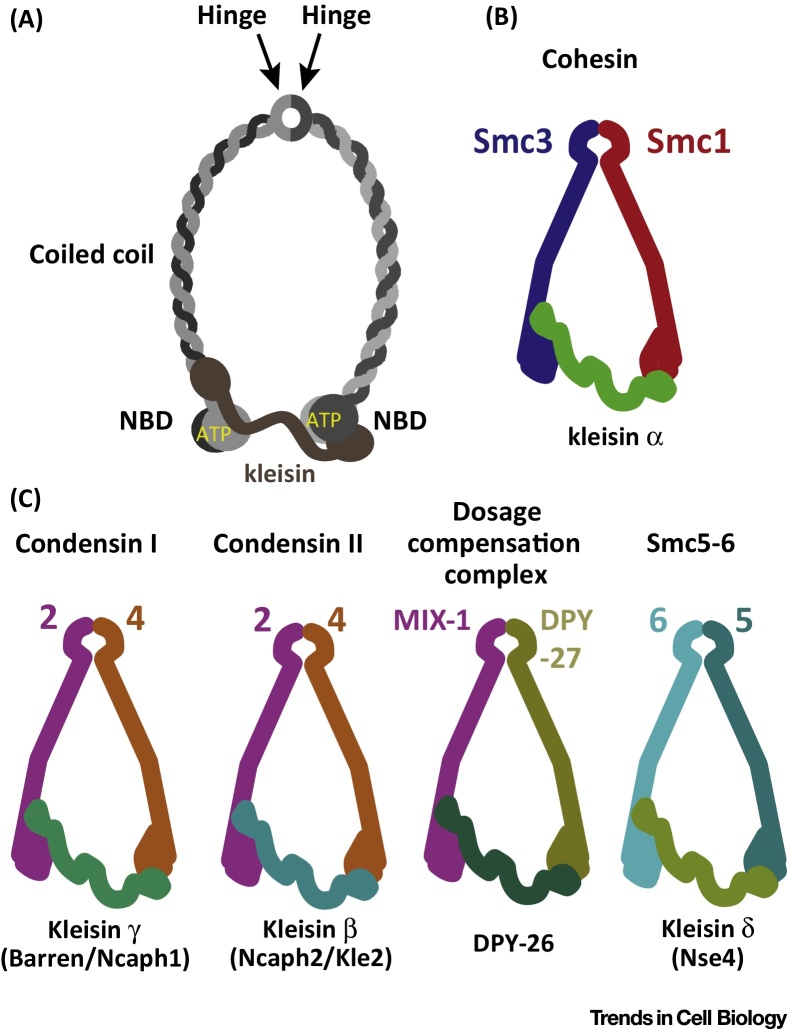
Structure and Variations of Structural Maintenance of Chromosomes (Smc)-Kleisin Molecules. (A) Domains of a typical Smc-kleisin complex. (B) The cohesin Smc-kleisin trimer. (C) The families of Smc-kleisins. Of note are the variations within condensins. The Nse4 kleisins likely comprise a δ-kleisin group. Abbreviation: NBD, nucleotide-binding domain.

Every cell division is accompanied by the segregation of two replicated sister genomes. From bacteria to Archaea and up to the most evolved eukaryotic metazoans, equal segregation of the chromosomal complement is mediated and secured by the extended family of Smc proteins [1]. Smc proteins share known basic features and are long, mostly helical polypeptides, typically exceeding 1000 residues in length. Smc proteins fold back on themselves at the hinge domain, forming an antiparallel coiled coil structure that extends over approximately 50 nm (Figure 1A). The N- and C-terminal domains of the polypeptide together form the globular nucleotide-binding domain (NBD).

The first step of complex formation is mediated by dimerisation through the hinge domains of two Smc proteins, forming a V-shaped dimer (Figure 1A). Each NBD binds one ATP molecule, which is hydrolysed when the catalytic pocket is complemented by residues found in an opposite site from the NBD of the other subunit. This sandwich arrangement of ATP binding and hydrolysis is shared with proteins of the ABC ATPase type and, indeed, Smc NBDs share sequence and structural homology with these proteins 2, 3, 4. In all known Smc complexes, Smc dimers form a heterotrimeric complex when bridged by a third subunit known as kleisin, at their NBD domains, which closes the V formation [5].

In eukaryotes, the primordial *Smc* gene has evolved through duplication and speciation into a range of orthologues and paralogues [3] (Figure 1B,C). Four trimeric Smc-kleisin complexes have been identified to form: (i) cohesin, which holds sister chromatids together from S phase to anaphase 6, 7, 8, 9, 10, 11; (ii) condensin I and condensin II, which are major determinants of chromosomal density and elasticity 12, 13, 14, 15; (iii) the dosage compensation complex, which is a variation of condensin that is involved in heterochromatin formation (Figure 1B,C) [16] (this complex is not present in all species and has mainly been studied in *Caenorhabditis elegans*, where the MIX1-DPY27 SMC heterodimer interacts with DPY-26 kleisin [17]); and (iv) Smc5-6 complex, which is involved in replication fork resolution and DNA repair 18, 19. The function of Smc5-6 goes beyond the architectural and structural role typically exhibited by cohesin and condensin since, in contrast to the peripheral subunits of both cohesin and condensin, the respective Smc5-6 Non-Smc Elements (Nse1–6) have enzymatic activities 20, 21, 22, 23. This complex is known to be essential for viability in yeasts [24] and for DNA repair in yeasts, invertebrate, and vertebrate model systems 25, 26, 27.

Given the overall size and presumed shape of the Smc-kleisin molecules, it became obvious that mechanistic solutions to questions regarding their function would only come from using rigorous structural and biochemical studies. Several crystal structures of SMC-related complexes have been solved recently, providing insight into their function. In this review, we focus on recent structural studies of the eukaryotic Smc1–6 members, their kleisin partners, and their regulators in hope of providing a picture of their mechanisms in chromosome segregation (progress on prokaryotic Smc proteins is summarised in [28])

## Cohesin and Establishment of Sister Chromatid Cohesion

During DNA replication and due to the torsional nature of the process, newly born DNA fibres are intertwined at sites where replication forks collide [29]. This catenation however is not efficient enough to permanently hold sister chromatids together. In a process tightly integrated with the cell cycle, sister chromatid cohesion is mediated by cohesin. Below we discuss how cohesin forms and associates with DNA, and how binding of cohesin to DNA mediates sister chromatid cohesion. Below, we discuss how cohesin forms and associates with DNA, and how binding of cohesin to DNA mediates sister chromatid cohesion.

### Cohesin: A Hoop to Trap Sister DNA Molecules

**Figure 2:**
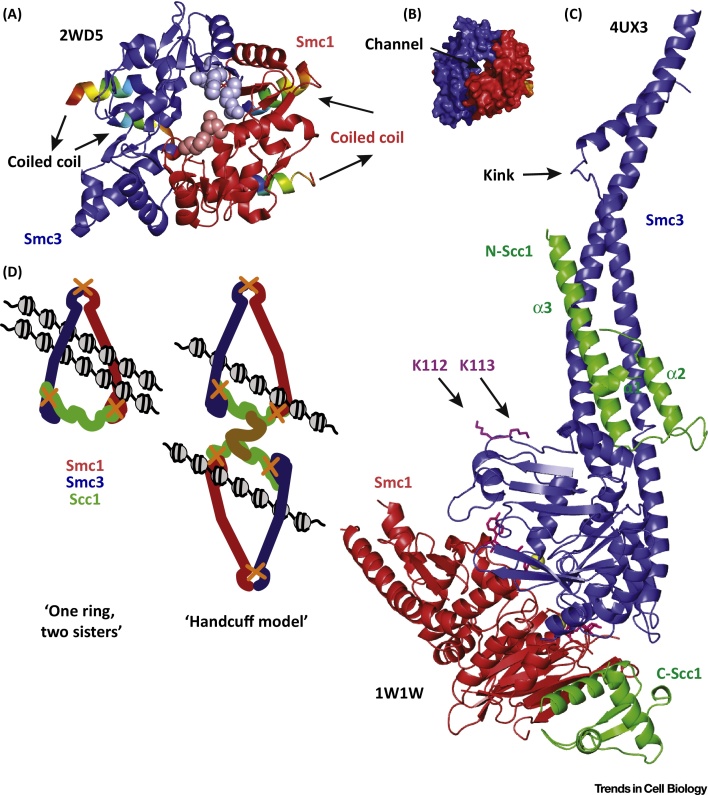
Structure of Three Cohesin Interfaces and Ring Formation. (A) Ribbon diagram of the *Mus musculus* structural maintenance of chromosomes 3 (Smc3; blue) and Smc1 (red) hinge interface [Protein Data Bank (PDB) 2WD5] seen from above. The entering and exiting helices of the coiled coils can be seen (rainbow colours, from blue to red following residue sequence). The side chains of the R665/K668/R669 to Alanine (Smc3) residues (light-blue spheres) and K554/K661 to Aspartate (Smc1) residues (light-blue spheres) mutated in the budding yeast charge removal experiments. (B) Surface diagram of the *M. musculus* hinge interface, as seen from above with the central channel. (C) Ribbon diagram of the yeast Smc3-kleisin (blue-green) interface (PDB 4UX3) combined with the yeast Smc1-kleisin (red-green) interface (PDB 1W1W). The ATP-binding pocket can be seen with ATP and Mg^+2^ bound (yellow sphere). The three helices of the N-terminus Scc1 and the winged helix motif of the C-terminus Scc1 interact asymmetrically with the Smcs. The acetylation patch (magenta, residues K112, K113) is distant from the Smc3-kleisin interface. (D) *In vivo* crosslinking data corroborate the simpler ‘one-ring, two sisters’ hypothesis rather than the ‘handcuff model’.

The heterodimer of Smc1 and Smc3, combined with the Scc1/Mcd1 (Rad21 in animals) α-kleisin subunit or its meiotic counterpart Rec8, form cohesin, which holds sister chromatids together from S phase to anaphase. The Smc1–3/Scc1 kleisin interface forms a tripartite structure resembling a ring 30, 31 (Figure 1B). The so-called ‘ring hypothesis’ dictates that one or more of the three interfaces of the cohesin complex serve as a topological entry gate for DNA and, once trapped inside the complex, a mechanism exists to ensure that sister chromatids cannot exit under tension produced by the metaphase spindle [32]. In budding yeast, the Smc1-Scc1 interface [33] comprises the most C-terminal domain of Scc1 and the NBD globular domain of Smc1 (Figure 2C, red and green). The NBD of Smc1 is similar in structure to previously obtained prokaryotic homologues 34, 35 and the C-terminal domain of Scc1 is a winged helix fold. The structure of the mouse cohesin hinge interface [36] (Figure 2A) resembles the bacterial homodimeric hinge structure from *Thermotoga maritima* [30]. The Smc1–3 mouse hinge crystal structure contains the globular domains and parts of the emerging helices of the Smc1 and Smc3 coiled coils. A channel in the middle gives the heterodimeric structure a doughnut shape (Figure 2B). The residues within this channel form a positively charged cavity, suggesting that it is a DNA-binding site during hinge interface opening for the loading of cohesin onto DNA [37]. However, mutations designed to reverse or neutralise these charges in budding yeast (Figure 2A, spheres) did not alter the binding of cohesin to yeast chromatin. However, they did affect the dissociation rate of the monomers and reduce the acetylation of Smc3 on the NBD, 50 nm away from the hinge when fully stretched, suggesting that hinge dissociation, possibly primed by interactions with other regulators, regulates the establishment of cohesion at S phase by an allosteric mechanism.

Completing the cohesin ring, the Scc1 N-terminal domain interacts with the coiled coil emerging from the NBD of Smc3, but not with the bottom surface of the NBD itself [38] (Figure 2C). Using chemical crosslinking and mass spectrometry, the Smc3-kleisin interface in human cohesin was also found to be located on the emerging coiled coil region of Smc3 [39]. Stressing the evolutionary conservation of these complexes, binding is similar to the interaction of Mre11 with Smc-like Rad50 protein 40, 41, 42 and of the bacterial Smc with the N-terminal domain of its ScpA kleisin [43]. In the Smc3 structure, the coiled coil extended beyond the Scc1 interaction interface and showed a pronounced break of the helical fold (Figure 2C, blue), demonstrating that interruptions seen in the computational coiled coil predictions of Smc lead to breaks in the double-helical coiled coil fold 44, 45. It has been suggested that kinks within the Smc coiled coil arms, often visible in electron microscopy (EM) images 30, 39, 46, are caused by these interruptions and have been suggested to mediate an interaction of the hinge with the ATPase 47, 48. The apical part of the NBD domain of Smc3 appears similar to that of Smc1. However, for the first time, it was possible to identify a sequence stretch containing two lysine residues (K112 and K113), where Smc3 is acetylated by the Eco1 acetyltransferase 49, 50, 51, found some distance from the Scc1 interface (Figure 2C).

Solving the structure of all three interfaces allowed testing of the ring model *in vivo* with thiol-specific cross-linking to entrap yeast circular mini-chromosomes [38]. Previous models have suggested that two cohesin complexes trap the two sisters either separately (handcuff model) or as a bigger hexameric complex [32]. However, circularised cohesin rings entrapped not only single chromosomes, supporting the two-ring ‘handcuff model’ of entrapment 52, 53, 54, but also a high abundance of two sister DNA molecules. This result corroborates the ‘one ring, two sisters’ hypothesis (Figure 2D) whereby a single cohesin trimer entraps both sister chromatids, because a prediction of the handcuff model is that only single chromosomes would be entrapped within the cohesin ring, whereas this result showed that sister chromatids were also entrapped in high amounts. In addition, the hexameric complex model could not be supported because all entrapping rings contained only one molecule of the SMC3 subunit [38]. Furthermore, recent studies in bacterial Smc complexes and yeast condensins support ring formation and topological functions for all Smc-kleisin complexes tested to date, reinforcing evolutionary connections between all Smc complexes 55, 56.

#### The Loading and Releasing Activities of Cohesin

**Figure 3:**
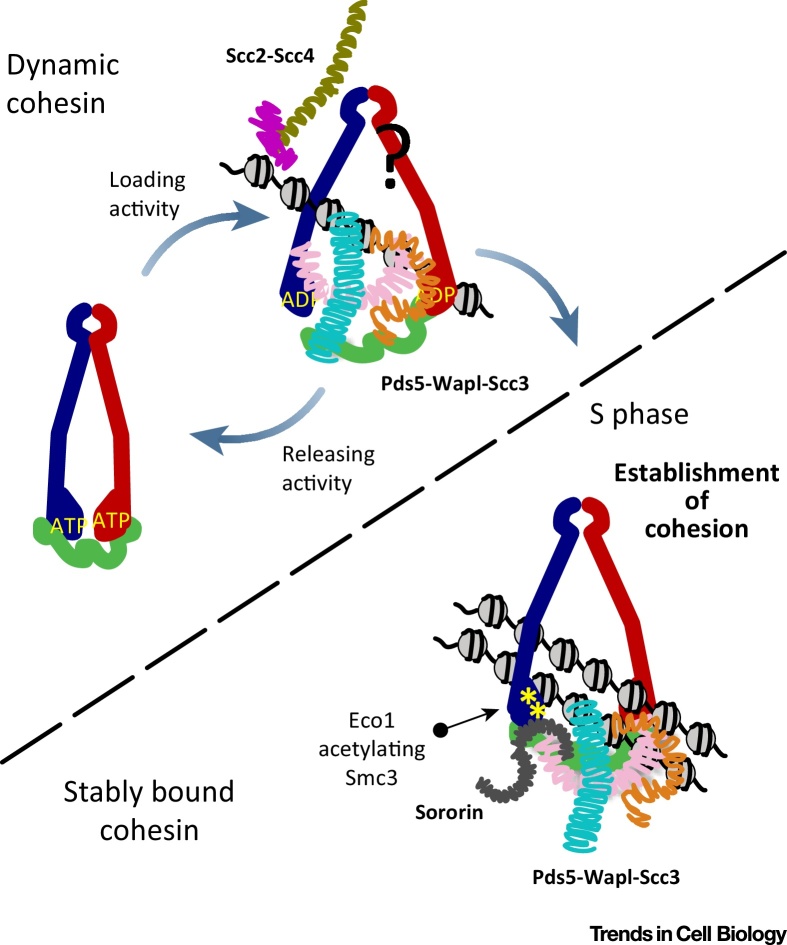
Cohesin Regulators and the Dynamic Life of Cohesin in the Nucleus. (A) Two main activities determine the turnover of cohesin on chromatin: Scc2-Scc4 (Nipbl-Mau2 in mammals) loads cohesin, while Pds5-Wapl-Scc3 releases cohesin from DNA. In S phase, the acetylation of structural maintenance of chromosomes 3 (Smc3) in two lysines of the nucleotide-binding domain (NBD) by Eco1 (Esco1/2) counteracts releasing, assisted by Sororin, a specific inhibitor of Wapl found in vertebrates. The cartoons are hypothetical.

Cohesin trimeric rings are destined to entrap DNA; however, the establishment and maintenance of sister chromatid cohesion depends on several peripheral proteins acting upon cohesin and eventually determining its residence time on chromatin. Proteins with essential functions in sister chromatid cohesion have been categorised based on the stage of their action and are involved in loading cohesin on DNA, establishing cohesion during S phase, or maintaining cohesion during mitosis (Figure 3). The Scc2-Scc4 complex (Nipbl-Mau2 in higher eukaryotes) has been implicated in the initial recruitment of cohesin onto chromatin 57, 58, the Eco1 acetyltransferase (Esco1/2) in establishing cohesion during S phase 50, 51, the Scc3 (SA1/2/3) subunit in both loading and cohesion maintenance 58, 59, 60, and the Pds5 (Pds5A/B) subunit in cohesion establishment and maintenance 61, 62, 63, 64, 65.

Since not all cohesin loading events will produce sister chromatid cohesion, a process must exist to remove this noncohesive population of rings from chromatin. The discovery of the so-called ‘prophase pathway’ in higher eukaryotes [66] came to refine the way we think about the release of cohesin from DNA. At the start of mitosis, cohesin is removed from chromosome arms in vertebrate cells through a dedicated cohesin ‘releasing activity’ mediated by Wapl (Wpl1/Rad61 in yeasts), while at the end of mitosis, cohesin is removed through proteolytic cleavage of the Rad21/Scc1 kleisin. Wapl acts in synergy with the Scc3/SA and Pds5 subunits, forming a subcomplex 67, 68, 69, 70 that displaces cohesin from DNA in all model organisms tested. Thus, Scc3 and Pds5, which were previously thought of as cohesion-promoting factors, have an additional role in releasing cohesin from DNA, implying that single subunits do not have single functions but instead are part of a larger machine that performs other functions when its parts are engaged in distinct conformations.

With this in mind, the dynamics of cohesin in the cell appear more complicated than previously anticipated (Figure 3). At certain time points, cohesin exists as two distinct populations: a dynamic population of measurable turnover on chromatin and a population stably bound to DNA with essentially no turnover. The dynamic population is a result of two opposing activities: (i) a DNA-loading activity mediated by Scc2-Scc4 and Scc3 that acts on the hinge interface (the most likely the DNA entry gate 37, 71); and (ii) a releasing activity executed by the Pds5-Wapl-Scc3 complex that affects the Smc3-kleisin interface, the putative DNA exit gate, and releases cohesin from DNA 39, 71, 72, 73. During S phase, Smc3 is acetylated by Eco1 at tandem lysine residues found on the surface of its ATPase domain (K112 and K113 in budding yeast), counteracting the releasing activity of the Pds5-Wapl-Scc3 complex. The position of the Smc3-Scc1 DNA exit gate some distance from this acetylation patch (Figure 2C) prompts the important question of how this double acetylation has such a crucial role in cohesion maintenance.

Collectively, these findings converge on the idea that Pds5, Scc3, and Wapl act together to both maintain cohesin on, and release it from, DNA. Thus, all three of these proteins have been the focus of a structural approach aimed at gaining mechanistic insight in these processes

#### Wapl: A Wedge Splitting the Smc3-Kleisin Interface?

**Figure 4:**
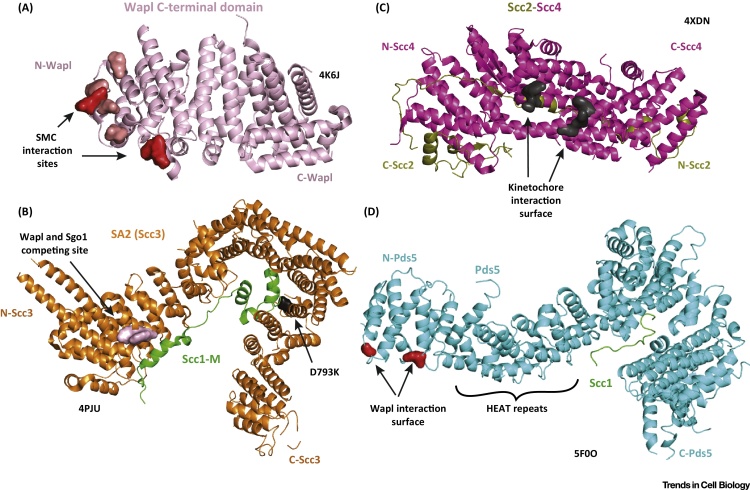
Structure of the Three Components of the Cohesin-Releasing Complex. (A) Ribbon diagram of the *Homo sapiens* C-terminal Wapl crystal structure [Protein Data Bank (PDB) 4K6J]. Wapl (pink) comprises entirely Huntington, Elongation Factor 3, PR65/A, TOR (HEAT) repeats. A surface patch important for releasing (light red), residues mediating the interaction with the cohesin ring (red), and residues with an uncharacterised effector are highlighted. (B) Structure of the *H. sapiens* Scc3/SA2 (orange) interacting with the middle region of the *H. sapiens* Scc1-kleisin (green). A two-helix protrusion emerges from the region that Wapl and Sgo1 compete over for binding (pink). A hot spot for Scc1 binding (black surface) was determined using the D793K side chain charge reversion. (C) Ribbon diagram of the crystal structure (PDB 4XDN) of *Saccharomyces cerevisiae* Scc4 (magenta) interacting with the first 131 residues of Scc2 (N-Scc2, olive). Scc4 forms 13 tetratrico peptide repeats (TPR) repeats, while the middle part of N-Scc2 crosses through a channel formed by the middle domain of Scc4. A surface patch interacting with a yet unknown kinetochore component is highlighted (black). (D) Ribbon diagram of the crystal structure of the *Lachancea thermotolerans* Pds5 (5F0O). A small peptide of Scc1 (*Lt* Scc1 121–143) is shown as a green coil. A patch highlighting homologous residues corresponding to previously identified suppressors of Eco1 deletion in *S. cerevisiae* (most likely interacting with Wapl) are highlighted in red.

The above findings established Wapl as a key contributor to DNA-releasing activity. In budding yeast, Wapl (Wpl1) determines the turnover of cohesin on the yeast point kinetochores, the latter being the major cohesin-loading site on every chromosome [58]. Loss of Wapl reduces the turnover of cohesin on chromatin 67, 73, 74 and affects chromosome compaction 75, 76. Two structures of Wapl derived from yeast *Ashbya gossypii* (AgWapl) and human (HsWapl) were recently solved 69, 77 (Figure 4A, HsWapl) and both revealed the globular and C-terminal half of the protein 69, 77. In both structures, Wapl contains eight Huntingtin, Elongation Factor 3, PR65/A, TOR (HEAT) repeats forming two distinct domains. An additional crystal structure of AgWapl contained a short Smc3-derived peptide that was suggested to correspond to an interaction region [77]. However, the observed binding region on HsWapl was not found to be necessary for any interaction with cohesin [69] and structural alignment with the recently obtained Smc3-Scc1 structure [38] suggests steric hindrance, incompatible with the hypothesis that Smc3 interacts with Wapl at this site.

Despite being crystallised with no interacting proteins, functional analysis assisted by the HsWapl structure revealed important regions for the function of Wapl [69]. The N-terminal half of Wapl (1–600; not present in the crystal structures and probably not folded) mediated the interaction with both Scc3 (region 500–580) and Pds5 (residues 1–450), whereas a region in the crystallised C-terminal part interacted with the trimeric Smc/Scc1 ring (region 647–684). Importantly, neither the N- or C-terminal half of Wapl was as efficient in binding cohesin as the full-length protein.

#### Scc3/SA2: A Large HEAT Repeat Protein Involved in the Prophase-Releasing Pathway

The most dramatic manifestation of the release of cohesin from DNA is the prophase/prometaphase pathway found in vertebrates [66]. At the onset of mitosis, most of the cohesin loaded on chromosome arms is removed by a wave of releasing activity that strips cohesin off the chromatin. Even so, a significant pool of cohesin at centromeres (and at their close proximity) remains intact and functional [66]. This cohesive population keeps sister chromatids attached 8, 78 and confers their biorientation and eventual alignment of the chromosomal complement at metaphase [79]. Thus, the obvious questions arising are: what is the effector removing cohesin from the arms and what is protecting centromeric and pericentric cohesin from this removal?

One model explaining this phenomenon relies on the finding that phosphorylation of Scc3/SA2 of cohesin, most likely by the mitotic kinase Plk1, destabilises cohesin on chromosomal arms, while the localised action of the Sgo1-PP2A phosphatase complex near kinetochores counteracts the destabilising phosphorylation, protecting centromeric cohesin from being released 80, 81, 82, 83. In agreement with this model, a version of SA2 that cannot be phosphorylated resists mitotic catastrophe that would normally follow Sgo1 depletion, suggesting that Sgo1-PP2A acts to keep a local pool of dephosphorylated SA2 available. However, the finding that mitotic SA2 can be found phosphorylated at kinetochores [84] challenges this idea and opens alternative possibilities of how the Sgo1-PP2A phosphatase complex protects centromeric cohesin.

Answers to these questions could result from the recently determined structure of Scc3 from the budding yeast *Zygosaccharomyces rouxii* [60] and its human SA2 counterpart with the interacting part of human Rad21 kleisin [85]. Due to high sequence similarity, the two Scc3 structures can be aligned and are similar. Both proteins are entirely helical, forming many tandem HEAT repeats (Figure 4B, HsSA2:Scc1/Rad21). In both cases, the HEAT repeats bend into a compact horseshoe shape. The inner concave surface of SA2 binds a large fragment of the middle part of Rad21 (Scc1-M, kleisin). That part of Rad21 comprises four distinct helices, interrupted by unstructured coil regions. Despite the fact that the SA2 surface involved in this interaction is highly conserved, finding single or multiple mutations that affect the interaction has been difficult, due to the large area on SA2 covered by Rad21 and the fact that Rad21 is significantly extended when binding. However, one such crucial residue was identified in SA2. D793K blocked the SA2–Rad21 interaction *in vitro* and resulted in premature sister chromatid separation in human cells (Figure 4B).

Obtaining the SA2–kleisin interaction presented a good opportunity to analyse in mechanistic detail the prophase pathway and the protection of centromeric cohesin. By using the available SA2-kleisin structure, an Sgo1-binding site was identified on SA2 and was found to functionally overlap with a neighbouring Wapl-binding site (Figure 4B). Purified Wapl and Sgo1 proteins competed for binding to SA2-Rad21, corroborating a hypothesis by which the Sgo1-PP2A phosphatase complex protects centromeric cohesin by sterically antagonising Wapl binding to cohesin. In support of this hypothesis, mutant versions of SA2 that do not bind Wapl did not require the function of Sgo1 [84]. Thus, the exact mechanisms by which centromeric cohesin is protected appear more complex than previously anticipated. Even if the model supporting a steric hindrance executed by Wapl is correct, the additional requirement of the dephosphorylase activity of Sgo1-PP2A indicates that both processes could be in action.

#### Pds5: A Gatekeeper with a Double Life

Both Scc3/SA and Pds5 are large helical proteins bearing HEAT repeats and both bind to the Scc1/Rad21 kleisin subunit 63, 86. However, Scc3/SA binds closer to the C-terminal domain of the kleisin (and, thus, closer to the stable Smc1-kleisin interface), while Pds5 binds proximally to the N-terminal domain of the kleisin and, thus, close to the Smc3-kleisin exit gate. While both Scc3/SA and Pds5 are necessary for the removal of cohesin from chromatin, both proteins are also essential for the maintenance of sister chromatin cohesion. The mechanism that causes Scc3 and Pds5 to switch from ‘anticohesion’ to ‘procohesion’ is a key issue.

Recent structural work using two closely related yeast species verified the placement of Pds5 in proximity of the Smc3-Scc1 interface. Recently, the structure of the bigger part of Pds5 from *Lachancea thermotorelans* (Figure 4D) was solved, in addition to the structure of the N-terminal half of Pds5 from *Saccharomyces cerevisiae* 87, 88. In both cases, small peptides of the respective Scc1 kleisin were crystallised with Pds5, designed based on previously published studies determining the interface using deletion analysis and photo-crosslinking [63]. The Pds5–kleisin interaction was shown to be essential for the promotion and protection of Smc3 acetylation [63], and the newly solved crystal structures reaffirm these findings. Furthermore, detecting the location of previously identified mutations in Pds5, which suppressed the loss of Eco1 in *S. cerevisiae*, revealed that these mutations collide and form a patch on the surface of Pds5 (Figure 4D). Importantly, an *in vitro* binding assay that combined purified Smc3, Scc1, and Pds5 proteins captured the formation of a trimeric complex. However, using small-angle X-ray scattering, the authors concluded that this trimer does not adapt to a unique confirmation due to the weak binding activity of Pds5 towards the Smc3 coiled coils. Thus, in both studies the kleisin peptide used for crystallography allowed the capture of this crucial interface and the interrogation of Pds5 function in cohesion maintenance and DNA release. In retrospect, however, it becomes clear that in-depth mechanistic analysis will necessitate the capture of structures of substantially bigger complexes.

#### The Scc2-Scc4 (NIPBL-MAU2) Cohesin Loader

Cohesin is believed to be recruited to chromatin through the action of the Scc2-Scc4 (Nipbl-Mau2) complex 57, 58. However, this idea has been challenged recently and, at least in yeast, the recruitment of both cohesin and Scc2-Scc4 onto chromatin appears to be interdependent [89]. Budding yeast Scc2 and Scc4 form a tight complex that is thought to interact with the kinetochore, the major loading site for yeast cohesin [58]. In addition, Nipped-B, the *Drosophila melanogaster* homologue of Scc2, was found to have a role in transcriptional regulation, a function potentially unrelated to cohesion 90, 91. The mammalian homologue Nipbl is mutated in more than 50% of cases of Cornelia de Lange syndrome, the most prominent of cohesinopathies 92, 93. Patients with this syndrome bear no chromosome segregation abnormalities, which implies that the cause of the disease is a malfunction beyond the role of cohesin and its loader in sister chromatid cohesion, reinforcing the idea of Scc2 and cohesin function in transcriptional control.

The N-terminal part of Scc2, interacting with the full-length Scc4 protein (the NScc2-Scc4 complex) was recently crystallised from the yeasts *Ashbya gossypii* [94] and *S. cerevisiae* [95], two close relatives. Consequently, the solved structures are similar (Figure 4C). Scc4 forms an almost entirely helical fold comprising 13 tetratrico peptide repeats (TPRs). Three Scc4 subdomains can be distinguished, the middle one delineating a channel where the central part of the Scc2 fragment is bound. Deletion of the interacting domains in yeast cells causes lethality and affects the recruitment of Scc2 to both kinetochores and chromosome arms [95]. When mutated, a conserved patch of residues found on the Scc4 surface (Figure 4C) affects specifically the recruitment of cohesin and Scc2 to yeast kinetochores, implicating this region in a as yet uncharacterised interaction with a presumed kinetochore component [95]. Using the full-length Scc2-Scc4 budding yeast complex and EM [94], images were obtained in which the Scc2-Scc4 complex adopts an extended S-like shape with Scc4 at one end forming a globular head and the extended HEAT motifs of the C-terminal Scc2 (a part missing from the crystal structures) at the other end forming a hook-like structure, resembling the conformation of Scc3 both in size and shape. Overall, these recent structural findings elucidated aspects of the recruitment of Scc2-Scc4 on kinetochores. Still, Scc2 remains the most enigmatic of all the regulatory subunits despite the fact that its function most likely is a functionally unique and biochemically distinct step in the complex interaction of cohesin rings with DNA. Thus, further biochemical and structural studies are necessary to gain mechanistic insight into the ways in which Scc2 functions.

## Condensin: From Rods and Butterflies to Elastic Chromosomes

From the earliest studies on condensin, its significance as a structural component of eukaryotic chromosomes was clear 96, 97. The heterodimer Smc2-4 interacts with either γ-kleisin Ncaph1 or β-kleisin Ncaph2 to form condensin I and condensin II, respectively. However, recent biochemical, structural, and cell-imaging studies have provided unparalleled mechanistic insight into the function of condensin. The conditional proteolytic cleavage of the mouse condensin II kleisin subunit (Ncaph2) in cells arrested in meiosis I produced dramatic decompaction of chromosomes under tension from the meiotic spindle [15]. By contrast, the formation of condensed chromosomes could be recapitulated *in vitro* using a minimum of six factors, along with DNA: histone octamers, histone chaperones, topoisomerase II, and condensin I [98]. The FACT nucleosome remodeller complex was also found to be essential for achieving chromosomal structures resembling properly condensed mitotic chromosomes. How condensin is mechanistically coupled to chromatin and to the FACT complex is an exciting new field of research. Again, the geometry of the condensin complex might dictate how these functional interactions occur.

Recent structural work 43, 99 provided additional insights into the shape of both the hinge and the emerging coiled coils of budding yeast Smc2-Smc4 condensin [100]. In previous rotary-shadowing EM images of Smc complexes, three main forms were observed: open, V-shaped, semi-open Y-shaped, and closed I-shaped (rod-like) 30, 46, 59. In most atomic structures, the coiled coils emerging from the two hinge monomers are either too short or point into opposing directions, indicating a V-shaped conformation [36] (Figure 2A). In a recently obtained structure, in addition to the globular domains of Smc2-Smc4, approximately 60 residues of the Smc2 and 120 residues of the Smc4 coiled coil were resolved (Figure 4D). The coiled coils are in close proximity, prompting the idea that, in addition to an open V-shape conformation, a closed conformation with the coiled coil segments running side-by-side can be adopted. This is supported by negatively stained EM images of the bacterial *Bacillus subtilis* Smc-ScpA trimer, where the Smc-kleisin trimers appear closed and rod shaped [36]. In addition, the structure of the archaeal *Pyrococcus furiosus* Smc hinge with approximately 60 residue-long coiled coils shows differences in the orientation of the emerging coiled coils but an overall side-by-side coiled coil arrangement is still present. Using thiol-specific crosslinking, the close proximity of the yeast Smc2-Smc4 coiled coils was verified in immunoprecipitated yeast condensin complexes.

How do these data reconcile with the evidence supporting a ring for cohesin and condensin? It seems likely that distinct conformations exist at different times of the functional cycle of these complexes. For example, in an *in vitro* assay using thiol-specific crosslinking, full-length bacterial Smc molecules abolished their rod shape when both DNA and ATP were present, again making a hinge–NBD allosteric interaction likely [100]. Such dynamic behaviour was confirmed in a recent study using purified Smc2-Smc4 dimers from budding yeast and high-speed atomic force microscopy (AFM) in liquid conditions [101]. Surprisingly, three novel condensin molecule conformations were observed: O-forms (corresponding to ring-shaped heterodimers), B-forms (corresponding to both heads interacting with the hinge and assuming a butterfly-like shape), and P-forms (with only one head interacting with the hinge). These abundant conformations covered 75% of the population, while the remaining 25% were found in the open V shape. However, no I-shaped rod-like dimers were observed, raising the concern of whether conventional EM, crystallography, and *in vitro* crosslinking experiments might be capturing either artificial or transitory states. The dynamic behaviour of Smc2-Smc4 heterodimers was tracked in real time and conformational transitions, primarily from O- to B-shapes, were observed. Even though the kleisin subunit was not present to determine the behaviour of condensin rings, these experiments do point towards the notion that condensin complexes are likely more flexible and dynamic in nature than previously anticipated.

## The Enigmatic Smc5-6 Complex: Closer to the Eukaryotic Common Ancestor?

Recently, the analysis of available structures of the Nse1-Nse3 subunits of the Smc5-6 complex [102] led to the discovery of a novel conserved class of kleisin-interacting proteins termed the ‘kleisin-interacting tandem winged-helix elements’ (kite) family. Kite proteins form homo- or heterodimers in bacteria and eukaryotes, respectively and contain tandem winged helix motifs (WH). The WH motifs mediate both the dimerisation of the monomers and the interaction with the kleisin. While kite orthologues were not identified as any of the known cohesin and condensin subunits, there is good structural similarity with prokaryotic ScpB and MukE kleisin partners. The MAGE family of tumour suppressors [21] also appears as a group of rapidly evolving paralogues, structurally similar to ScpB and Nse1-Nse3 kites.

Why does Smc5-6 maintain kite protein interactions whereas cohesin and condensin do not? The interesting hypothesis arising is that Smc5-6 potentially represents the closest eukaryotic relative to a common Smc ancestor. Based on this model, cohesin and condensin eventually lost their kite dimers while Smc5-6 retained them. An essential remaining question is whether the Nse5-Nse6 subunits of Smc5-6 are orthologues of the HEAT repeat subunits of cohesin and condensin (i.e., of Pds5-Scc3/SA and CapD2-CapG, respectively). A definitive answer to this question is not possible given the lack of relevant structural information for Nse5-Nse6. Another possibility could be that Nse5-Nse6, which operate as DNA recruiters during DNA repair and form a dimer 103, 104, functionally resemble the Scc2-Scc4 loader of cohesin rather than Scc3/SA and Pds5.

Irrespectively, an interesting implication of the emergence of the kite family is that the common eukaryotic ancestor likely performed DNA maintenance, cohesion, and condensation functions. Hence, the dedicated and chromatin-related cohesion and condensation processes could represent an evolutionary refinement of the more general entrapment activity. This could also explain why Smc5-6 is not generally essential 105, 106, because cohesin has been found to be essential for DNA repair 107, 108, keeping the two sister DNAs together during the repair process [109].

## Concluding Remarks

**Figure 5:**
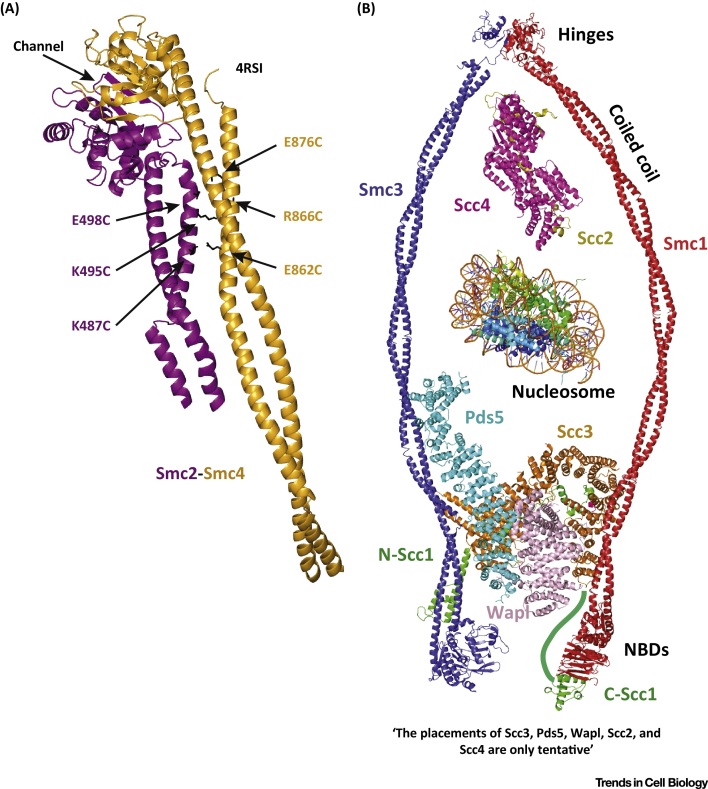
Structure of the Yeast Condensin Hinge and Coiled Coils. (A) Ribbon diagram of the crystal structure (PDB 4RSI) of the budding yeast structural maintenance of chromosomes 2 (Smc2)-Smc4 hinge with extended coiled coil domains (purple-orange). The hinge channel is almost perpendicular to the axis of the coiled coil. Residues and respective side chains used to demonstrate coiled coil contacts using thiol specific crosslinking are highlighted. (B) A combination of the known cohesin structures compiled based on known or proposed topologies. A yeast nucleosome has been added in the near background. Pds5, Scc3, and Wapl are placed at the bottom of the cohesin ring based on structural evidence. Scc2-Scc4 has been proposed to act closer to the hinge. The coiled coil domains are modelled, while all other ribbon diagrams are of published structures.

Recent progress in the structural biology of the Smc complexes, especially in the case of cohesin, has helped us to better understand the distinct roles of different subunits, domains, and various regulators of the interactions of Smc complexes with DNA and chromatin. Over the next few years, we anticipate that cryoEM, cryotomography and single molecule-imaging methods, such as smFRET, will take over as the most important methods for characterising the various holocomplexes (Figure 5B), because many components now have good atomic models. For example, visualisation of the entrapment of DNA is still outstanding and we believe that EM will be able to deliver mechanistic details regarding DNA folding within cohesin rings. We hope that it has become clear that only a combination of genetics, imaging, biochemistry, reconstitution experiments, and structures will be able to move us forward (see Outstanding Questions). Some promising first steps towards such efforts have been taken both for condensin and for cohesin 47, 98. The Smc5-6 complex remains mysterious, and might well be a harder problem mainly because it appears to act as an enzyme with a plethora of targets rather than as a structural element.

An even bigger question to answer will be how these machines that shape chromosomes and dictate chromatin functions functionally interact with other factors, such as the CTCF pioneer binding factor in mammals 110, 111, 112. Cohesin appears to have adopted a noncanonical function (i.e., a role beyond sister chromatid cohesion) in mammals by entrapping DNA segments of the same chromosome. The end result of this function is the creation of looped chromatin fibres. In this way, cohesin might prove to be a central player in 3D genome structuring and be involved even more in genome reforming during cell differentiation and tissue formation 113, 114, 115, 116. This noncanonical function of cohesin could be beneficial in maintaining genomic stability and fine-tuning transcriptional activity [117]. The significance of such a role for nuclear homeostasis is becoming obvious with the ever-increasing involvement of cohesin in developmental disease and cancer 118, 119, 120. We believe that the mechanistic progress made studying the canonical role of cohesin should become imperative for further research on the recently identified noncanonical role of cohesin in animals.

Outstanding Questions What is the most basic, fundamental function that makes Smc complexes ubiquitous?

What is the precise series of events that leads to loading, entrapment, releasing, and stable cohesion?

What is the exact role of the NBD domains? How does ATP binding and hydrolysis affect the loading and releasing processes?

Are there similar processes, such as loading and releasing activities, in condensin and Smc5-6?

What is the precise architecture of the head complex (NBDs, kleisin, Scc3, Wapl, and Pds5)?

Is there any long-range communication between the NBDs and the hinges?

What are the structures of the Smc5-6 kleisin complexes? Is Nse4 a bona fide kleisin?

How does CTCF direct DNA looping by cohesin and what is the topology of those complexes?

Is there a direct mechanistic interplay between different Smc-kleisin complexes in organising chromatin?
